# Dysfunctional Prefrontal Function Is Associated with Impulsivity in People with Internet Gaming Disorder during a Delay Discounting Task

**DOI:** 10.3389/fpsyt.2017.00287

**Published:** 2017-12-13

**Authors:** Yifan Wang, Yanbo Hu, Jiaojing Xu, Hongli Zhou, Xiao Lin, Xiaoxia Du, Guangheng Dong

**Affiliations:** ^1^Department of Psychology, Zhejiang Normal University, Jinhua, China; ^2^School of Psychology and Cognitive Science, East China Normal University, Shanghai, China; ^3^Department of Psychology, London Metropolitan University, London, United Kingdom; ^4^School of Psychology, Southwest University, Chongqing, China; ^5^Peking-Tsinghua Center for Life Science, Peking University, Beijing, China; ^6^Department of Physics, Shanghai Key Laboratory of Magnetic Resonance, East China Normal University, Shanghai, China; ^7^Institute of Psychological and Brain Sciences, Zhejiang Normal University, Jinhua, China

**Keywords:** Internet gaming disorder, decision-making, delay discounting task, dorsolateral prefrontal cortex, inferior frontal gyrus

## Abstract

Internet gaming disorder (IGD), defined as the persistent use of online games with ignorance of adverse consequences, has increasingly raised widespread public concerns. This study aimed at elucidating the precise mechanisms underlying IGD by comparing intertemporal decision-making process between 18 IGD participants and 21 matched healthy controls (HCs). Both behavioral and fMRI data were recorded from a delay discounting task. At the behavioral level, the IGD showed a higher discount rate *k* than HC; and in IGD group, both the reaction time (delay − immediate) and the discount rate *k* were significantly positively correlated with the severity of IGD. At the neural level, the IGD exhibited reduced brain activations in the dorsolateral prefrontal cortex and bilateral inferior frontal gyrus compared to HC during performing delay trials relative to immediate ones. Taken together, the results suggested that IGD showed deficits in making decisions and tended to pursuit immediate satisfaction. The underlying mechanism arises from the deficient ability in evaluating between delayed reward and immediate satisfaction, and the impaired ability in impulse inhibition, which may be associated with the dysfunction of the prefrontal activation. These might be the reason why IGD continue playing online games in spite of facing severe negative consequences.

## Introduction

Internet gaming disorder (IGD) has increasingly raised widespread public concerns. It is defined as recurrent and persistent use of online games, which lead to a variety of negative consequences in terms of daily life and mental health, such as maladaptive coping, ill interpersonal relationship, and decreased academic achievements (1, 2). Experimental studies and questionnaire surveys have indicated that individuals with IGD show great behavioral and neuronal similarities to those with drug addictions, substance abuse, and gambling disorder in many aspects, involving comorbid psychiatric symptoms, behavior control, and decision-making (3–5). Nevertheless, compared with substance-related and addictive disorders (e.g., alcohol abuse disorder), a significant feature for IGD is no substance or chemical intake. In May 2013, IGD has been listed in Section “Results” of the DSM-5 as a condition warranting further studies (6–8).

Intertemporal decision-making refers to situations where people need to choose between two options: an immediate but smaller reward and a delayed but larger one (9). Delay discounting task (DDT) is a widely used paradigm in exploring intertemporal decision-making and measuring impulsive choices (10), but rarely used to detect the decision-making and planning of IGD. When the delay is shorter, people generally prefer the larger reward rather than the smaller one; but with gradually increased delay, people will shift their preference to the smaller reward rather than the larger one. Individuals who shift their preferences to smaller rewards after shorter delays would be regarded as more impulsive than individuals who shift their preferences after longer delays (11). Studies using DDT have found that delayed rewards tend to be more steeply discounted in substance addicts in relation to alcohol (12), heroin (13), cocaine (14), methamphetamine (15), and pathological gamblers (16) when compared to healthy controls (HCs). Furthermore, there is evidence that individuals with IGD are more impulsive than recreational Internet gaming users and HC (17–20). These findings raise the possibility that the IGD, in accordance with drug and gambling addicts, show myopia for the future, i.e., preference for short-term rewards (e.g., Internet games) and ignorance for long-term losses (e.g., social relationship).

Previous works with the DDT established the neural correlates of brain regions in intertemporal decision-making and then proposed a dual-valuation model, which assumed that there were two separate systems contributing to such decisions (21, 22). One system (called the “β system”) included mesolimbic dopamine projection regions and weighed the immediate rewards (i.e., nucleus accumbens and medial prefrontal cortex); the other system (called the “δ system”) included the lateral prefrontal cortical areas and weighed the delayed rewards. Human imaging studies also explored brain activations during delay discounting process in behavioral addiction and substance dependence samples. Pathological gamblers showed elevated brain activities in the dorsolateral prefrontal cortex (DLPFC) and amygdala when selecting delayed rewards compared with HC (23). Alcoholics were reported to show increased activities in the inferior frontal gyrus (IFG), insula, and supplementary motor area along with steeper discounting of delayed rewards (24). Smokers also exhibited dysfunctional brain activations in the IFG, DLPFC, and insula during the inhibition of immediate smaller rewards to gain the delayed larger ones (25). The DLPFC has been proved to be involved in behavioral inhibition, reward processing, and decision-making; the IFG is also critical for inhibition and risky decision-making; besides, the insula plays a part in cognitive function and motor control (26–28). Specifically, the altered functional connectivity in the bilateral prefrontal lobe has been detected in IGD (29).

Although previous researches have revealed decision-making deficits in IGD, the underlying mechanism of impaired ability to control their behaviors remains unclear. To explore the reasons why individuals with IGD pursue instantaneous rewarding experience regardless of long-term benefits, 21 HCs and 18 IGD were recruited to perform the DDT, which comprised a series of selections between immediate smaller monetary rewards and delayed larger monetary rewards.

Our previous study has found that the participants with IGD were prone to take risks and exhibited less activation in the IFG and superior temporal gyri when making risky choices in comparison to HC (30). A study that used Go/No-Go paradigm with gaming cue distraction found that the IGD showed impaired response inhibition and decreased brain activities in the right DLPFC (31). In individuals with IGD, viewing Internet gaming-related stimuli significantly induced increased brain activations in the prefrontal cortex, inferior parietal lobule, and striatum (19, 20, 32). These findings suggest that the brain regions associated with cognitive control, craving, decision-making, and reward induce dysfunctional effects by virtue of the frequent use of Internet games in IGD. Therefore, we hypothesized that the IGD group may show similar behavioral tendency (myopia for the future) and brain activation patterns parallel with findings in other addiction disorders. At the behavioral level, we expected to observe steeper discounting of delayed rewards in IGD compared to HC and a modulation of delayed reward representations by the severity of IGD. At the neural level, we expected IGD to show less brain activations in those brain regions (i.e., DLPFC, IFG), which are related to the evaluation of delayed rewards, and to impulse inhibition. We also expected that brain activations would be correlated with behavioral performances in IGD group.

## Materials and Methods

### Participants

The experiment conforms to the Code of Ethics of the World Medical Association (Declaration of Helsinki). The Human Investigations Committee of Zhejiang Normal University approved this research. All participants signed the informed consent forms before the experiment. Participants were right-handed male students (18 IGD and 21 HC) recruited through advertisements in Shanghai, PR China. Only males were included due to higher IGD prevalence in men than that in women. There were several exclusion criteria for selecting participants, including history or current neurological or mental disorders as measured by MINI international neuropsychiatric interview and the mood states scale, history or current psychiatric disease (e.g., depression, schizophrenia), and history of drug abuse (e.g., cocaine, alcohol) or any other type of behavioral addictions as measured by standard interviews and self-report instruments. All participants did not report a history of behavioral addiction, substance abuses, and mental disorders. Importantly, none of them reported brain injuries, brain surgeries, and any attention problems such as attention deficit hyperactivity disorder. In addition, all participants were told to not take any addictive substances 3 h before the experiment began, including coffee, cigarette, and alcohol.

The diagnosis of IGD was determined based on (1) a modified Young’s online Internet Addiction Test (33), which emphasized on IGD (IAT, see Supplementary Material), (2) the proposed nine-item IGD diagnostic scale based on DSM-5 (34), and (3) the criteria for time and frequency of gaming playing. Both the questionnaire and criteria were precisely translated into Chinese for the suitability of participants. To critically assess gaming behaviors and IGD symptoms, we then replaced all the statements of online activities in the original questionnaire with specific items, such as game playing or online games. The validity of the modified IAT was tested, and the Cronbach’s alpha coefficient of reliability index was an acceptable 0.90. The modified IAT consists of 20 items associated with online games including psychological dependence, compulsive use, withdrawal, related problems in school or work, sleep, family, and time management. For each item, participants were instructed to choose a number from the following scale: 1 = “Rarely” to 5 = “Always,” or “Does not Apply.” The score of the modified IAT is ranged from 20 to 100, which represents the severity of IGD. Scores over 50 indicate occasional or frequent Internet addiction problems, and scores over 80 indicate severe Internet addiction problems (35).

The demographic characteristics for both groups were shown in Table 1. The IGD and HC did not significantly differ in age and education years. In this study, the IGD group was composed of individuals who (1) scored over 50 on the modified IAT, (2) met at least five of the nine DSM-5 criteria, (3) spent at least 2 h on online games per day during the last 2 years, and (4) spent most of their online time playing online games (>80%). However, the HC group did not satisfy any above-mentioned criteria.

**Table 1:** Demographic characteristics for HC and IGD participants.

	HC (M ± SD)	IGD (M ± SD)	*t*	*p*
Age	23.1 ± 2.0	22.1 ± 3.2	1.2	0.25
Years of education	14.6 ± 1.4	14.4 ± 1.6	0.8	0.42
IAT	31.5 ± 11.9	64.0 ± 10.1	9.1	0.00**
DSM	1.3 ± 0.9	5.2 ± 0.8	12.1	0.00**
Time spent on games per day (in hours)	0.5 ± 0.2	2.9 ± 0.4	23.6	0.00**

HC, healthy control; IGD, Internet gaming disorder. ** p < 0.01.

### Task and Procedure

**Figure 1:**
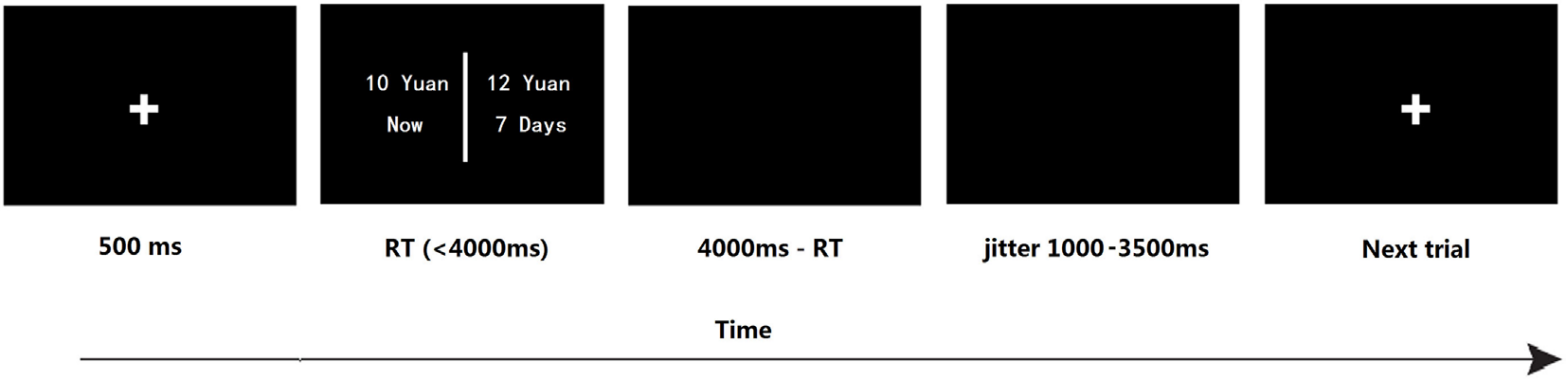
The timeline of one trial in the delay discounting task. The immediate but smaller option is fixed on 10 Yuan; in the delayed but larger options, monetary amounts ranged from 12 to 15, 20, 30, 40, and 50 Yuan, and the delay time ranged from 6 h to 1, 3, 7, 30, and 90 days. “Yuan” is the basic unit of money in China.

The whole time of the task lasted about 15 min for each participant. Participants first practiced 20 trials to be familiar with the task before completed the DDT task in the scanner. During the task, participants need to make choices between an immediate reward and a larger amount of money with a specified delayed time (e.g., now 10 Yuan versus 7 days later 12 Yuan, $1 is equal to about 6.6 Yuan). The monetary amounts varied from 12 to 15, 20, 30, 40, and 50 Yuan, and the delay time ranged from 6 h to 1, 3, 7, 30, and 90 days. Thus, there were 36 trials in 1 block, and the task consisted 2 blocks in total. The trials in this study were presented randomly in E-prime (version 2.0, Psychology Software Tool, Figure 1).

All participants were paid a guaranteed 40 Yuan (≈$6) for the participation and an extra reward (ranged from 12 to 50 Yuan) that depended on their selections in DDT task. To elicit participants’ motivation to response properly, they were informed that they would receive additional payments according to their performances during the task. For example, if they selected the fixed money on the trial, then they would gain 10 Yuan in cash; if they selected the delayed option, they would gain that amount of money in cash after the corresponding delay.

### Behavioral Data Analysis

Delay discounting rate was estimated for each participant by the following hyperbolic model (36):

(1) $$V=\frac{A}{\left(1+kD\right)}.$$

The *V* represents the subjective value of the delayed reward; *A* is the amount of the delayed reward; *D* is the length of delay to its delivery; and *k* is a free parameter that indicates the steepness of the discount curve. Higher *k* values indicate more rapid discounting and greater impulsivity (37–39). An important procedure for estimating *k* value was to determine the indifference points, which were the points that the fixed reward and the delayed reward were of equal subjective value for an individual. The indifference points were calculated across a series of different delay lengths and monetary amounts and were fitted into the Eq. 1. There were two steps of the behavior data analyses for DDT. In the first step, a non-linear curve-fitting program (Origin 7.0) was used to determine each participant’s best-fit values of *k*. The second step was to perform a log 10 transformation of the *k* values. The log transformation was required for these data due to their non-normal distribution (40, 41). To examine the different discount rate *k* of IGD and HC, an independent sample *t* test was performed.

### Image Acquisition and Pre-Processing

fMRI data were collected using a 3T scanner (Siemens Trio) with a gradient-echo EPI T2 sensitive pulse sequence in 33 slices (interleaved sequence, 3-mm thickness, repetition time = 2,000 ms, echo time (TE) = 30 ms, flip angle 90°, field of view 220 × 220 mm^2^, matrix 64 × 64). Stimuli were presented by *Invivo* synchronous system (*Invivo* Company)^1^ through a monitor in the head coil. Structural images covering the whole brain were collected using a T1-weighted three-dimensional spoiled gradient-recalled sequence (176 slices, flip angle = 15°, TE = 3.93 ms, slice thickness = 1.0 mm, skip = 0 mm, inversion time = 1100 ms, field of view = 240 × 240 mm, and in-plane resolution = 256 × 256).

The pre-processing of imaging analysis was conducted through Statistical Parametric Mapping (SPM) software package, SPM5.^2^ Images were slice-timed, reoriented, and realigned to the first volume. T1-co-registered volumes were then normalized to an SPM T1 template and spatially smoothed using a 6-mm full-at-half-maximum Gaussian kernel.

### First-Level Regression Analysis

A general linear model (GLM) was applied to identify blood oxygen level dependence (BOLD) signal in relation to two conditions: choice of immediate smaller reward and choice of delayed larger reward. Error trials were excluded. The GLM was independently applied to each voxel to identify voxels that were significantly activated for the event types of interest. A high pass filter (cut-off period = 128 s) was applied to improve the signal-to-noise ratio by filtering out low frequency noise.

### Second-Level Group Analysis

Second-level analysis was performed at the group level. First, we determined which voxels showed a main effect of delayed trials versus immediate trials within each group (IGD, HC). Second, we tested which voxels significantly differed in BOLD signal between IGD and HC [(IGD_delay_ − IGD_immediate_) − (HC_delay_ − HC_immediate_)]. Third, we identified clusters of contiguously significant voxels at an uncorrected threshold *p* < 0.05. Finally, we tested these clusters for cluster-level FWE correction *p* < 0.05, and the AlphaSim estimation indicated that clusters with 102 contiguous voxels would achieve an effective FWE threshold *p* < 0.05. The smoothing kernel was 6.0 mm, which was used during simulating false-positive (noise) maps through AlphaSim and was estimated from the residual fields of the contrast maps used in the one-sample *t*-test.

### Correlation Analysis

Correlation analysis was calculated between brain activities and the behavioral performances to test our hypothesis. We further carried out ROI analyses with seed regions from contrast delay trials versus immediate trials. For each ROI, a representative beta value was obtained by averaging the signal of all the voxels within the ROI. The correlations among the severity of IGD, log *k* values, reaction time (RT), and the beta values were calculated. The RT stands for the difference between the response to delayed options and the response to immediate options (delay – immediate).

## Results

### Behavioral Performance

**Figure 2:**
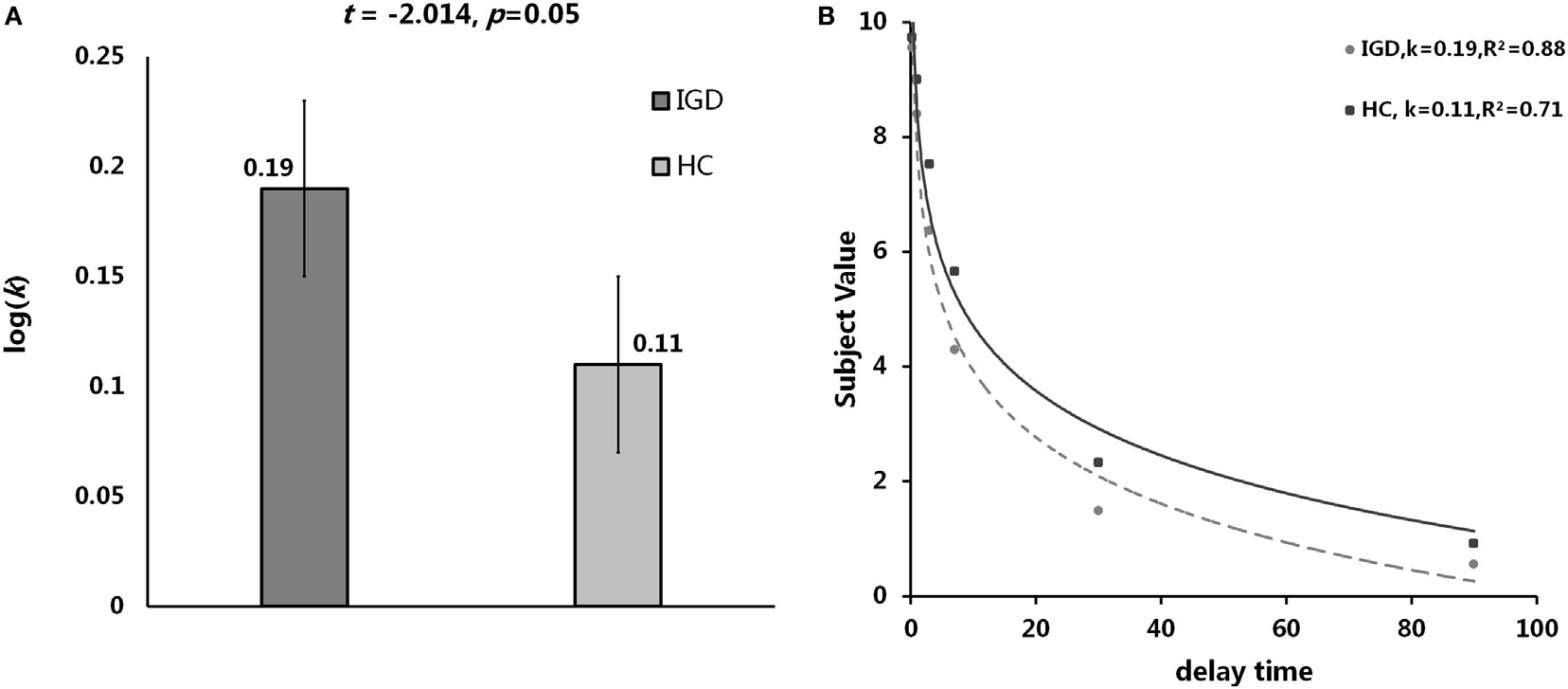
Delay discounting value differences between Internet gaming disorder (IGD) and healthy control (HC). **(A)** The IGD showed higher *k* value than HC. **(B)** Delay discount functions for HC and IGD. Points show mean indifferent points for monetary rewards as a function of delay time. *R*^2^ represents how close the fitted curve is from the actual data points. First, the variation between data points and the mean values is calculated. In least-squares fitting, the total sum of squares (TSS) includes two parts: the variation explained by regression and that not explained by regression [the residual sum of square (RSS)]. Then the *R*^2^ = 1 − RSS/TSS.

**Figure 3:**
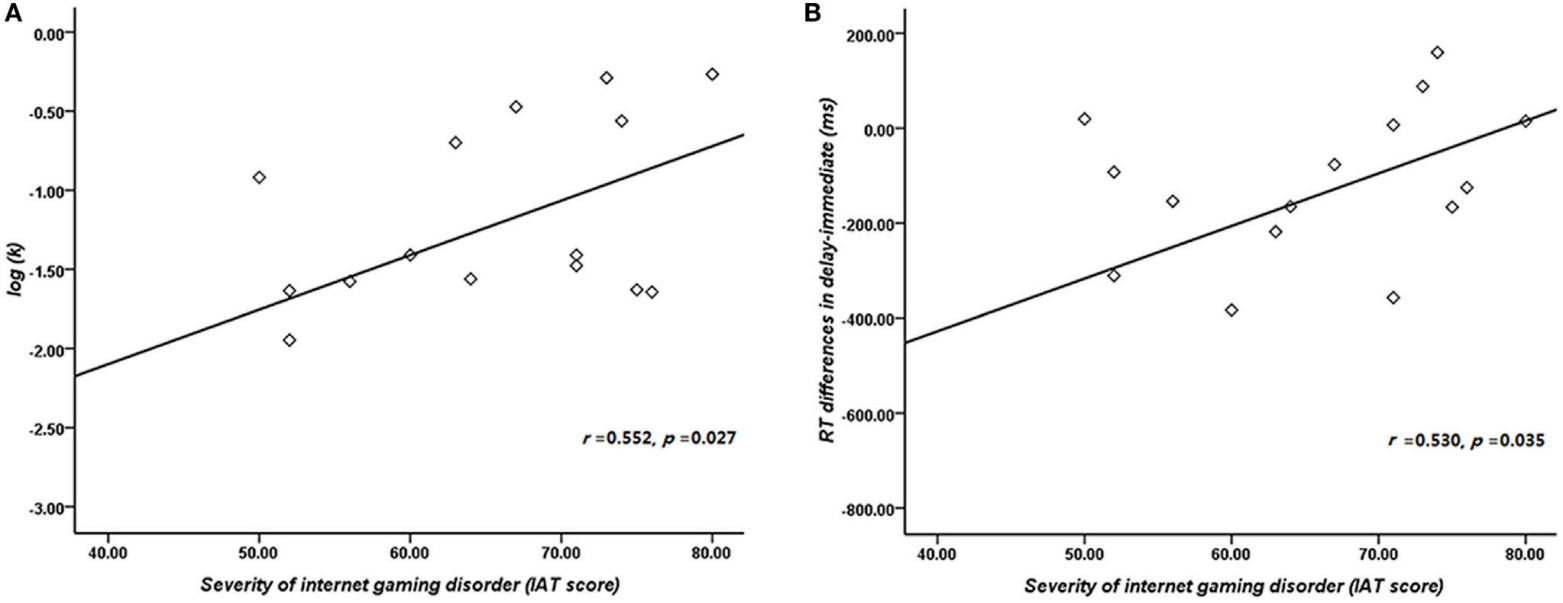
Correlation between the severity of Internet gaming disorder (IGD) and behavioral performance. **(A)** Correlation between the severity of IGD and log *k*. **(B)** Correlation between the severity of IGD and reaction time (delay − immediate). (Scores greater than 3 SDs were regarded as outliers and were excluded from further analysis.)

The result of independent sample *t*-test suggested that the *k* value of IGD was higher than that of HC at a marginal significant level (*t* = 2.01, *p* = 0.05, *d* = 0.53). The mean discounting rate *k* values and corresponding SDs for IGD and HC were 0.19 ± 0.16 and 0.11 ± 0.14, respectively (Figure 2A), and this indicated the IGD discounted the rewards more steeply than HC (Figure 2B). The *R*^2^ value for discounting function (0.88 for IGD and 0.71 for HC) denoted the variance accounted for by the Eq. 1. The RT (delay − immediate) of IGD was longer than HC, but it did not reach statistical significance (HC: −86 ± 213 ms, IGD: −56 ± 194 ms, *t* (1, 37) = 1.43, *p* = 0.11). In addition, the severity of IGD was significantly positively correlated with the log *k* values (*r* = 0.552, *p* = 0.027; Figure 3A) and RT (*r* = 0.530, *p* = 0.035; Figure 3B) in IGD group. But the correlations among these variables did not reach a significant level in HC group.

### Imaging Results

**Figure 4:**
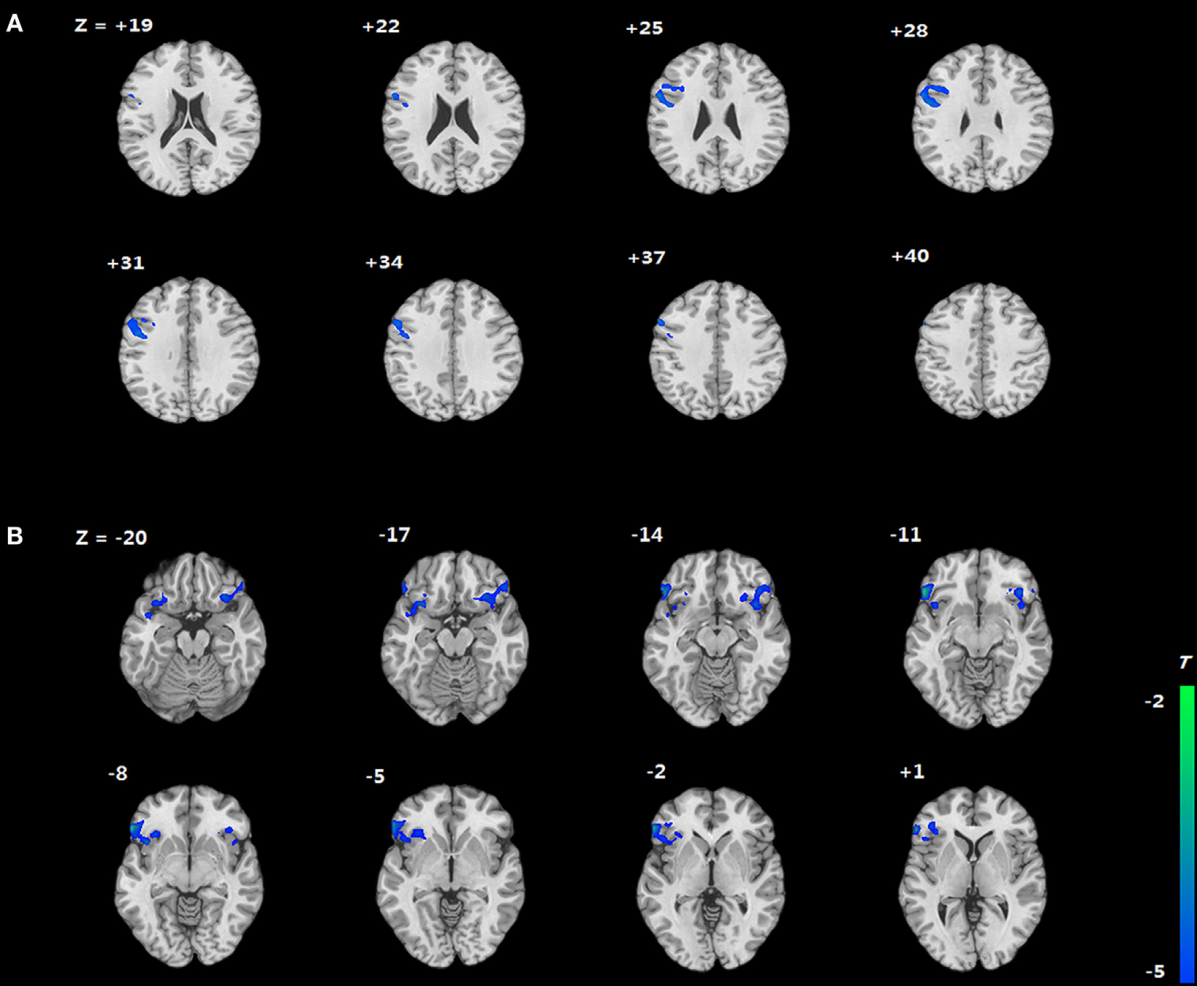
Brain areas showing differences in Internet gaming disorder (IGD) when comparing to healthy control (HC) [(IGD_delay_ − IGD_immediate_) − (HC_delay_ − HC_immediate_)]. **(A)** IGD show lower brain activation in left dorsolateral prefrontal cortex than HC. **(B)** IGD show lower brain activation in bilateral IFG than HC.

**Figure 5:**
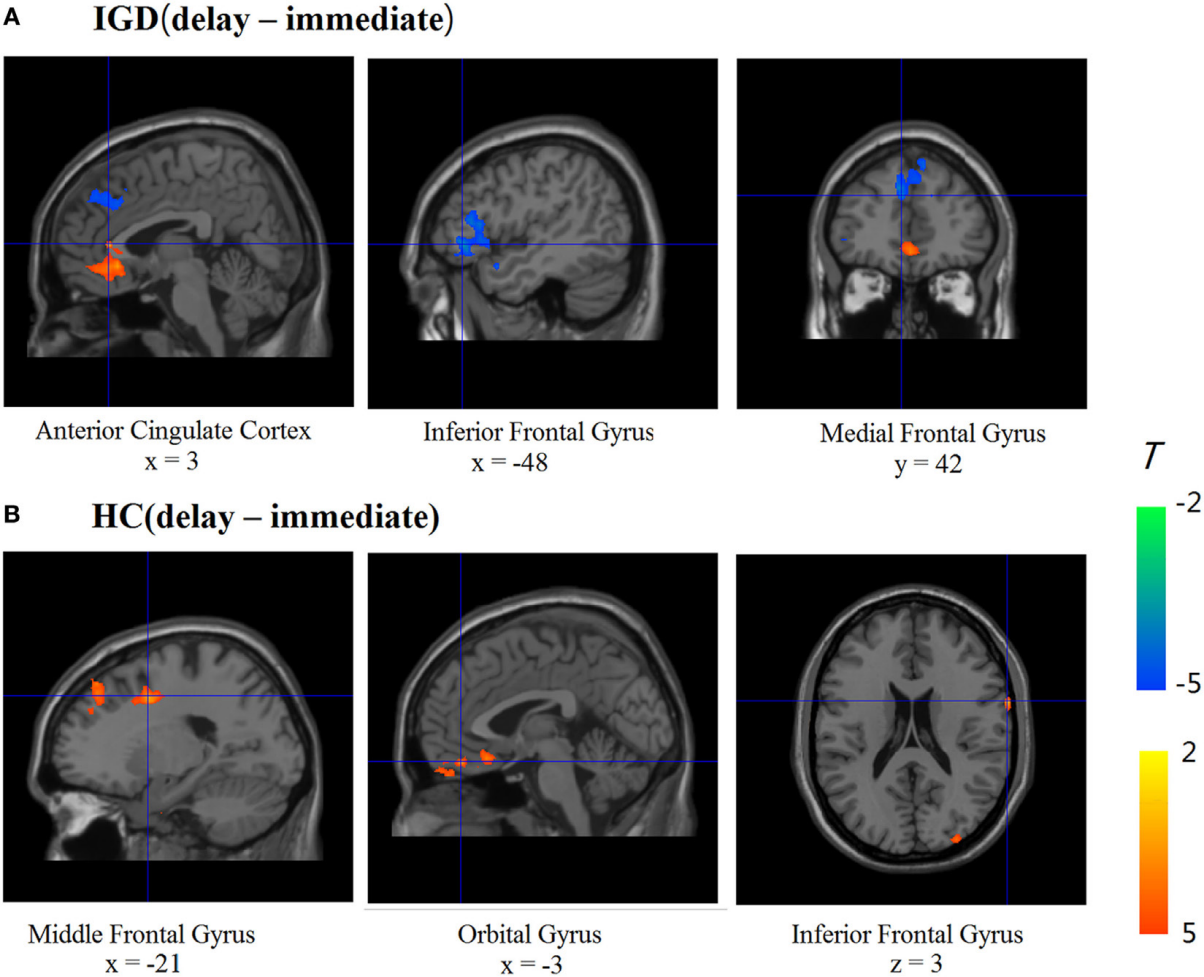
Brain activations change between different conditions in Internet gaming disorder (IGD) and healthy control (HC) (delay − immediate). **(A)** The IGD showed greater brain activation in the ACC and lower brain activations in the left inferior frontal gyrus (IFG) and medial frontal gyrus (delay > immediate). **(B)** The HC showed greater brain activations in the right IFG, orbital gyrus, and middle frontal gyrus (delay > immediate).

We compared the two groups in terms of BOLD signal differences between delayed choices and immediate choices. Group comparison suggested that the IGD showed smaller BOLD signal differences, between delayed and immediate choice, over the left DLPFC and bilateral IFG than HC (Figure 4 and Table 2), which was consistent with our hypothesis. Nevertheless, the IGD did not show any greater BOLD signals in the whole brain compared to HC. In each group, the IGD showed greater brain activations in the anterior cingulate gyrus and lower brain activations in the left IFG and medial frontal gyrus for delayed choices than immediate choices; the HC showed greater brain activations in the right IFG, orbital gyrus, and middle frontal gyrus for delayed choices than immediate choices (Figure 5 and Table 3).

**Table 2:** Brain activations change between IGD and HC (delay − immediate).

Region^a^	BA	*x*, *y*, *z*^b^	Max *t*	Number of voxels^c^	H
Inferior frontal gyrus	47	−54, 27, −12	−3.93	257	L
Dorsolateral prefrontal cortex	9	−57, 9, 24	−3.23	113	L
Inferior frontal gyrus	47	39, 27, −12	−3.02	109	R

*^a^The brain regions with maximal t score were selected to be shown*.

*^b^Peak Montreal Neurological Institute coordinates*.

*^c^Coordinates represent the local maxima in the delay > immediate contrast. If multiple local maxima existed in the same region, only the maximum with the highest t score is shown. Voxel size = 3 × 3 × 3*.

*HC, healthy control; IGD, Internet gaming disorder*.

**Table 3:** Brain activations change between different conditions in IGD and HC.

Region^a^	BA	*x*, *y*, *z*^b^	Max *t*	Number of voxels^c^	H
**IGD(delay − immediate)**					
Anterior cingulate cortex	24	3, 33, 6	5.74	198	R
Medial frontal gyrus	9	−6, 42, 27	−4.21	149	L
Inferior frontal gyrus	47	−48, 33, −6	−4.20	268	L
**HC(delay − immediate)**					
Inferior frontal gyrus	44	66, 21, 3	5.16	195	R
Orbital gyrus	11	−3, 42, −21	4.50	510	L
Middle frontal gyrus	6	−21, 3, 42	4.30	254	L

*^a^The brain regions with maximal t score were selected to be shown*.

*^b^Peak Montreal Neurological Institute coordinates*.

*^c^Coordinates represent the local maxima in the delay > immediate contrast. If multiple local maxima existed in the same region, only the maximum with the highest t score is shown. Voxel size = 3 × 3 × 3*.

*HC, healthy control; IGD, Internet gaming disorder*.

### Correlation Results

**Figure 6:**
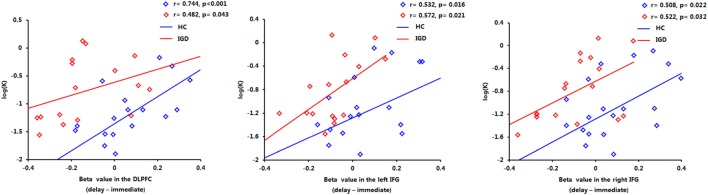
Positive correlations between the brain activations in the dorsolateral prefrontal cortex (DLPFC) and bilateral inferior frontal gyrus (IFG) and log *k* in both groups.

**Figure 7:**
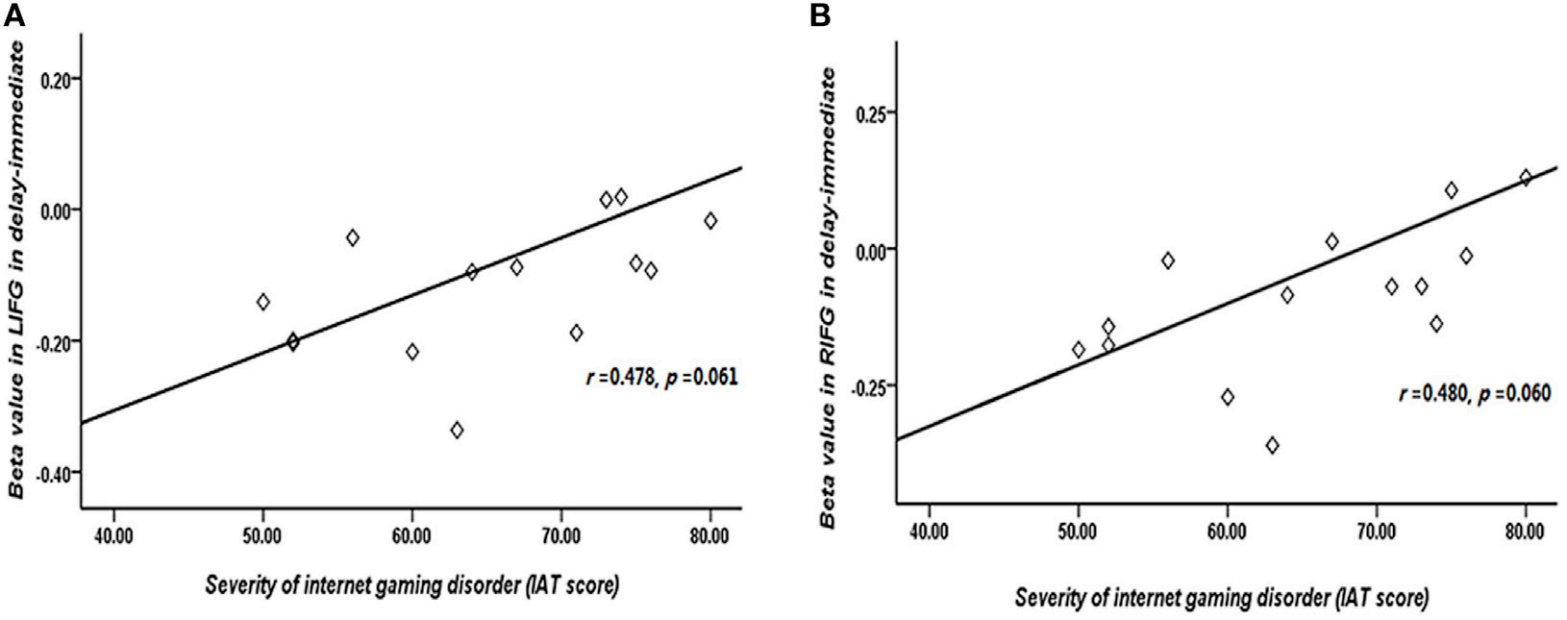
Correlation between the severity of Internet gaming disorder (IGD) and brain activations in the bilateral inferior frontal gyrus (IFG). **(A)** Correlation between peak left IFG activation (delay − immediate) and the severity of IGD. **(B)** Correlation between peak right IFG activation (delay − immediate) and the severity of IGD. (Scores that greater than 3 SDs were regarded as outliers and were excluded from further analysis.)

The correlations between beta values and behavioral performance were analyzed within each group. The brain activations in the DLPFC and bilateral IFG were all significantly positively correlated with the log *k* values in both groups (see the results in Figure 6), and the correlation between beta value in the DLPFC and log *k* in the two groups was significantly different by a Fisher’s *Z* test (*z* = 2.44, *p* < 0.05). In IGD group, the brain activations in the bilateral IFG (delay − immediate) were positively correlated with the severity of IGD, but it did not reach the significant level (left IFG: *r* = 0.478, *p* = 0.061; right IFG: *r* = 0.480, *p* = 0.060; Figure 7); no significant correlations were found between brain activations and the severity of IGD in HC group (*p* > 0.1). In addition, there were no significant correlations between the brain activations and RT in each group (*p* > 0.1).

## Discussion

Consistent with our hypotheses, the IGD showed higher discounting rate *k* and less brain activations than HC. The foregoing results indicated that the IGD group were more impulsive and might have deficient decision-making ability, which was in line with our previous study (42). In particular, we found that the left DLPFC and bilateral IFG were more deactivated in trials in which the IGD selected the delayed rewards compared to HC, which may provide evidences to further understand the mechanisms underlying IGD.

### Deficient Ability in Evaluating the Delayed Reward in IGD

Compared with HC, IGD showed lower brain activations in the left DLPFC when choosing the delayed options. Consistent with this finding, Hoffman et al.’s study found that methamphetamine-dependent individuals exhibited lower activation in the DLPFC than that of HC in delayed decisions (43). According to the dual-system mode, the δ system, which included the DLPFC, was mainly used for weighting the delayed rewards (21, 22). Researchers have also found that the DLPFC primarily responds to the delays of delayed rewards, and the activation in the DLPFC is negatively related to increasing delay time (44). Specifically, there is evidence that the DLPFC plays a vital role in encoding the attributes of multiple reward predictions into an integrated value (45).

Thus, the relatively reduced brain activities in the DLPFC observed in IGD may indicate that IGD had potential deficits in evaluating the magnitudes and delays of rewards. They could not fully integrate all the information of choices, which would lead to a lower capability in decision-making, even with longer decision-making time. Furthermore, a resting-state study has identified that the individuals with IGD show reduced functional connectivity strength between the DLPFC and caudate, suggesting impaired effective modulation of the DLPFC on rewards (46), which are also observed in substance abuse populations (47). Another explanation for the results is that there may be a minimum activation threshold of the DLPFC for individuals to choose the delayed reward. The activation below the minimum threshold would connect with the decisions for the immediate reward rather than the delayed one. Because the IGD have a lower activation of the DLPFC, they reach the minimum threshold at shorter delays than HC.

In addition, the RT was positively correlated with the severity of IGD, indicating that the more serious the IGD was, the longer time they needed to make choices. The correlation findings supported the explanation that the IGD showed deficient evaluating ability of the delayed features to some extent. To sum up, we inferred that the IGD unconsciously focused on the short-term gains, which might be associated with the poor reward evaluation ability.

### Impaired Impulse Inhibition in Decision-Making in IGD

Apart from for the known role in reward processing, the DLPFC, as the highest-order association area, is also responsible for executive functions such as response inhibition and multi-attribute decision-making (48, 49). Especially, studies have proved that the activity in the DLPFC will enhance when individuals exercise self-control (50). Moreover, reduced brain activation of the IFG was also observed in IGD during the inhibition processing in the present research. It has been noted that the IFG is involved in cognitive control and impulse inhibition (51, 52). Moreover, the IFG is responsible for self-control and inhibition of prepotent responses for giving up immediate gratification and seeking for long-term interests (53–55). Critically, the IFG has also been identified as a crucial structure in the process of establishing flexible association between outcomes and advantageous actions (56). In general, the DLPFC and IFG play essential roles in the deployment of self-control and impulse inhibition. In this study, the lower BOLD signal in the bilateral IFG and DLPFC may reflect that the impaired ability for the IGD to control their behaviors and inhibit their impulse.

The altered brain activities in the DLPFC and IFG have been reported in previous researches, which reveal the low capacity of impulse inhibition in response to immediate rewards in IGD. Probabilistic discounting task have detected that the IGD exhibited high level of impulsivity and diminished BOLD signal in the IFG than both HC and recreational gaming users (18, 57). During risky decision-making, the IGD showed altered modulation of the bilateral DLPFC when taking risky choices (58). Moreover, we also found that the brain activations in the DLPFC and bilateral IFG were positively correlated with the log *k* values, suggesting that the IGD with greater activation local to the DLPFC and IFG was more impulsive. Although attributed to extracognitive endeavor by the prefrontal activation, the IGD cannot effectively control themselves to choose the delayed reward in the selection process.

In addition, positive correlation was found between the severity of IGD and the log *k* values, suggesting individuals with IGD who showed more severe IGD symptoms were also more impulsive. Another positive correlation between the severity of IGD and brain activation in the bilateral IFG might indicate that the more severe the IGD was, the more endeavors they needed to engage in selecting delayed decisions. What’s more, impaired executive control and reward circuit have been detected in IGD (42), which is parallel with our findings. Taken all into consideration, the results suggested that the IGD demonstrated deficient ability in reward evaluation and impulse inhibition, which might be associated with the dysfunction of the prefrontal activation. These findings are consistent with a prior meta-analysis of fMRI studies, implicating that dysfunctional prefrontal activation plays an important role of in the neurobiological mechanism of IGD (59).

### Limitations

There were several limitations ought to be noted. First, only male participants were recruited in this study, thus further studies should shed light on female participants. Second, to ease the difficulty of the tasks and let participants concentrate on the decision-making process, we did not balance the positions of the immediate options and delayed options, which might potentially bias the results.

## Conclusion

In summary, this study suggested that IGD showed steeper discounting rate and altered brain activities in the DLPFC and IFG. The mechanism might lie in their impairment in both evaluating the delayed reward and impulse inhibition ability in decision-making, which was associated with the dysfunction of prefrontal function. This could be a reason why they prefer immediate satisfaction to larger delayed rewards. More broadly, our research findings also provide insights into the reason why IGD continue playing online games even when they are faced with severe negative consequences caused by excessive engagement in Internet games.

## Ethics Statement

The experiment conforms to The Code of Ethics of the World Medical Association (Declaration of Helsinki). The Human Investigations Committee of Zhejiang Normal University approved this research. All subjects signed the informed consent forms before the experiment.

## Author Contributions

YW contributed to experimental programming, data collection and data analyses and wrote the first draft of the manuscript. GD designed this research. YH and GD revised and improved the manuscript. JX, HZ, XL, and XD contributed to experimental programming, and data collection. All authors contributed to and have approved the final manuscript.

## Conflict of Interest Statement

The authors declare that the research was conducted in the absence of any commercial or financial relationships that could be construed as a potential conflict of interest.

## Footnotes

^1^http://www.invivocorp.com/.

^2^http://www.fil.ion.ucl.ac.uk/spm/.
