# Supplementary material for: Dysfunctional Prefrontal Function Is Associated with Impulsivity in People with Internet Gaming Disorder during a Delay Discounting Task
Source: Front Psychiatry. 2017 Dec 13;8:287. doi: 10.3389/fpsyt.2017.00287 (PMC5733533; doi:10.3389/fpsyt.2017.00287)
Supplement: Supplementary file 1 [file presentation_1.pdf]

The Diagnostic Questionnaire for Internet Gaming Disorder

Instruction: The following questionnaire consists of 20 statements. Please read the statement carefully and select an answer (from 1 to 5) that best describes your situation during the past month. 1 means ‘Never’, 2 means ‘Occasionally’, 3 means ‘Frequently’, 4 means ‘Often’, and 5 means ‘Always’. Please read all the statements carefully before making your choice.

1. How often do you find that you play online games longer than you intended?
2. How often do you neglect household chores to spend more time on online games?
3. How often do you prefer the excitement of the Internet to intimacy with your gaming partners?
4. How often do you form new relationships with other game players?
5. How often do others in your life complain to you about the amount of time you spend on online games?
6. How often do your grades or school work suffer because of the amount of time you spend on online games?
7. How often do you play online games before something else that you need to do?
8. How often does your job performance or productivity suffer because of the online games?
9. How often do you become defensive or secretive when anyone asks the time you spend on games?
10. How often do you block out disturbing thoughts about your life with soothing thoughts of the online games?
11. How often do you find yourself anticipating when you will play games again?
12. How often do you fear that life without the online games would be boring, empty, and joyless?
13. How often do you snap, yell, or act annoyed if someone bothers you while you are playing online games?
14. How often do you lose sleep due to playing online games?
15. How often do you feel preoccupied with the online games when off-line, or fantasize about being playing games?
16. How often do you find yourself saying "just a few more minutes" when you play games?
17. How often do you try to cut down the amount of time you spend on online games and fail?
18. How often do you try to hide how long you've played online games?
19. How often do you choose to spend more time on online games over going out with others?
20. How often do you feel depressed, moody or nervous when you are off-line, which goes away once you are back to games?
